# Gingival recession and associated factors in a homogeneous Mexican adult male population: A cross-sectional study

**DOI:** 10.4317/medoral.17815

**Published:** 2012-05-01

**Authors:** Mirna Minaya-Sánchez, Carlo E. Medina-Solís, Ana A. Vallejos-Sánchez, Maria L. Marquez-Corona, América P. Pontigo-Loyola, Horacio Islas-Granillo, Gerardo Maupomé

**Affiliations:** 1 DDS., MSc. DDS., MSc. Faculty of Dentistry, Autonomous University of Campeche, Campeche, Mexico; 2 DDS., MSc. DDS., MSc. DDS., MSc., PhD. DDS., MSc. Academic Area of Dentistry, Health Sciences Institute at Autonomous University of Hidalgo State, Pachuca, Hidalgo, Mexico; 3 DDS., MSc., PhD Indiana University, Purdue University at Indianapolis School of Dentistry. Indianapolis, IN, USA and The Regenstrief Institute. Indianapolis, IN, USA

## Abstract

Background: Diverse variables are implicated in the pathogenesis of gingival recession; more detailed knowledge about the relationship between the clinical presentation of gingival recession and assorted risk indicators may lead to improved patient monitoring, early intervention, and subsequent prevention. The objective was to evaluate clinically gingival recession in a homogeneous Mexican adult male population and to determine the strength of association with related factors. Method: A cross-sectional study was carried out in a largely homogeneous group in terms of ethnic background, socioeconomic status, gender, occupation, and medical/dental insurance, in Campeche, Mexico. Periodontal examinations were undertaken to determine diverse clinical dental variables. All periodontal clinical examinations were assessed using the Florida Probe System, a dental chair and one examiner. Questionnaires were used to collect diverse risk indicators. Statistical analyses were undertaken with negative binomial regression models. Results: The mean number of sites with gingival recession per subject was 6.73±5.81; the prevalence was 87.6%. In the negative binomial regression model we observed that for (i) each year of age, and (ii) each percentage unit of increase in sites with plaque, and (iii) with suppuration, mean sites with gingival recession increased 2.9%, 1.0% and 13.0%, respectively. Having a spouse was associated with gingival recession. Conclusions: We observed association between gingival recession, and sociodemographic and clinical parameters. Patients need to be educated about risk indicators for gingival recession as well as the preventive maneuvers that may be implemented to minimize its occurrence. The potential of improved oral self-care to prevent a largely benign condition such as gingival recession is important, given the associated disorders that may ensue root exposure, such as root caries and root hypersensitivity.

** Key words:**Oral health, periodontal health, gingival recession, adults, Mexico.

## Introduction

Increasing evidence linking poor oral health with diverse systemic health problems continues to be reported in the scientific literature, including cardiovascular diseases, respiratory diseases, cancer, nutritional problems, hypertension, and diabetes mellitus. Poor oral health may also have a negative impact upon quality of life; the World Health Organization has encouraged public healthcare administrators and decision makers to design effective and affordable strategies for better oral health and quality of life (1). Dental caries and periodontal diseases and their consequences affect a large number of people in Mexico, and are considered public health problems (2-4). Compared to dental caries, periodontal diseases have received little attention in Mexico (1). Periodontal health can be evaluated through different indicators including gingival recession. Gingival recession is the exposure of the root surface due to a displacement of the gingival margin apical of the cemento-enamel junction (5). Its etiology is determined by a number of predisposing and precipitating factors (6). Predisposing factors may be anatomical or associated with occlusal trauma. The anatomical include poorly adhered gingiva, tooth malposition and crowding, root prominence, and bone defects. Those associated with occlusal trauma are related to the intensity and duration of trauma. In contrast, precipitating factors are a series of sociodemographic, socioeconomic and environmental issues. For example, some studies have observed that gingival recession was associated with sex, number of teeth present, bleeding on probing (BOP), the presence or absence of systemic disease(s), use of dentures, and use of alcohol and tobacco (7), or with inflammation measurements such as presence of plaque (8). Pires et al. (9) reported that the presence of gingival recession in the anterior lingual mandibular region of a young population was associated with the use of piercings, age, male gender, and BOP. Chronic systemic diseases, such as the diabetes, are factors that increase gingival recession (10). The role of tooth brushing on gingival recession, caused either by abrasion or trauma, is inconclusive (11). To further complicate the scenario, the proportion of subjects who have gingival recession vary markedly between countries and age groups (12).

In addition to maintaining dental and periodontal health, dental esthetics has become a great concern for both dental practitioners and patients: the gingival plane, gingival outline, and gingival recession in anterior teeth are particularly important (13). From a clinical perspective, gingival recession is one factor that predisposes to root caries (7), undermines aesthetic profiles (14), and favors the initiation of tooth sensitivity (5,12). The objective of this study was to add to the wealth of international data on gingival recession by evaluating clinically a homogeneous Mexican adult male population in the context of associated factors.

## Material and Methods

This study complied with the specifications of protection of study participants and adhered to the ethical regulations of the School of Dentistry at Universidad Autónoma de Campeche, the state public university in Campeche, Mexico. Prior to any data collection, potential participants were briefed on the objectives of the study, their right to participate or not, the outcomes and benefits, and were invited to decide if they wanted to participate. Informed consent letters were obtained, and participants were informed of their oral health status.

Campeche is a coastal city and capital city of the state of Campeche in the eastern Gulf of Mexico, part of the Yucatan Peninsula littoral. Campeche is a seaport with tourism, fishing, lumber, and agricultural industries. Campeche is included in the nationwide domestic salt fluoridation program.

Study design and subject selection

This is a cross-sectional study undertaken in a non-probabilistic sample of police officers from the city of Campeche (Secretaría de Seguridad, Vialidad y Transporte, the city’s police force). This is a largely homogeneous group in terms of ethnic background, socioeconomic status, gender, occupation, and medical insurance. A description of the survey planning and methods has been previously published (15,16). Inclusion criteria were male gender, older than 20 years of age, and with at least 6 natural teeth. Exclusion criteria were edentulous individuals, individuals whose diminished ability to open the mouth impeded a clinical exam, individuals undergoing periodontal treatment, and individuals currently taking antibiotics; three subjects were excluded for any one of these reasons. After obtaining informed consent, the total sample was 161 male subjects (100% accepted to be included in the study).

Clinical examination and data collection

Clinical variables were collected by a periodontist using a computerized periodontal probe (Florida Probe® - Florida Probe Corp. Gainesville FL) (17). Such probe has electronically controlled probing pressure and standardized recording capabilities, thus accruing more reliable readings than manual probing. The instrument is equipped with a 0.45 mm diameter tip; its precision is 0.2 mm with a regulated pressure of 15 grams. We evaluated six sites (distobuccal, midbuccal, mesiobuccal, distolingual, midlingual, and mesiolingual) in each tooth present per subject, except third molars (a maximum of 168 sites/subject). Subjects were examined in a dental chair with a dental lamp.

The dependent variable was gingival recession; operationally, gingival recession was called when the gingival margin was at least 2 mm apical to the cement-enamel junction. When the gingival margin was coronal to the cemento-enamel junction, the values were recorded as negative. We also collected periodontal variables such as pocket depth (18); dental plaque using the modified Silness and Löe index which also conveyed information on BOP through the Florida Probe; suppuration was ascertained by finger pressure on each one of the sites and direct observation (presence/absence). All of those variables were further quantified by establishing their extent, i.e., the number of affected sites divided by the number of sites examined multiplied by 100. We also recorded the number of natural teeth present.

A questionnaire was administered to obtain non-clinical variables and oral hygiene habits including age, marital status, maximum level of education, frequency of tooth brushing, dental floss utilization, use of dental care in the previous year, to be undergoing dental treatment at the time of the examination, use of antibiotics in the previous six months, and tobacco and alcohol intake. Tobacco and alcohol intake were categorized similar to other studies (19). Those subjects who had never smoked were considered non-smokers. Those subjects who had never had consumed any type of alcoholic beverage were considered non-drinkers. Ex-drinkers and ex-smokers were subjects who had abstained from any type of drinking and smoking for at least six months. Current drinkers and smokers were subjects who had consumed frequently any type of alcoholic beverage and smoked at least one serving / one cigarette in the previous months.

Data analysis

Firstly, a univariate analysis was carried out to obtain summary measures according to the scale of each variable. Subsequently, bivariate and multivariate analyses were done using a model for count variables. In general, a common approach to analyze count variables is Poisson regression; it has been well established, however, that a limitation is placed with the expectation that the mean and the variance must be equal. Over dispersion undermines the approach in that the model may be well specified and the estimates of parameters may be appropriate, but standard errors could still be incorrect. This situation results in an overestimate of parameter values and a subsequent increase in the width of confidence intervals. Such potential problem has been addressed by means of different strategies in the literature, including resorting to negative binomial regression. Knowing that our data were affected by over dispersion, we selected the latter strategy in our analyses to ascertain the percentage of change expected in the mean number of sites with gingival recession. The final model was built using a backward fitting. To fit the model we incorporated those variables which had a value p<0.25 in the bivariate model, and excluded those which were shown to be non-significant.

Analyses were undertaken in Stata 9.0®.

## Results

A total of 161 subjects were examined and interviewed (mean age 38.3±10.9, all male). The univariate results are shown in ( Table 1 and  Table 2). The clinical variables are presented in ( Table 2). Mean number of teeth and sites examined per subject was 24.45±4.63 and 146.72±27.80, respectively. Mean teeth lost were 3.55±4.63. In terms of sites affected with various conditions, we found 23.51±21.72 % of sites with plaque; 0.72±1.69 % of sites with suppuration; 15.60±15.22 % of sites positive to BOP; and 5.94±8.08 % of sites with pocket ≥ 4 mm. With regard to dental visits, 91.9% of the policemen reported at least one within the past year (reasons for visits were not collected) and 21.1% stated that they were undergoing some sort of dental care at the time of the study. For behaviors, 93.8% and 18.6% reported that they brushed their teeth at least once a day and used dental floss regularly, respectively. Finally, 39.7% of participants reported tooth grinding. The mean number of sites with gingival recession per subject was 6.73±5.81, with a maximum of 25 sites affected. The prevalence was 87.6%; only 20 subjects presented no sites with recession.

**Table 1:**
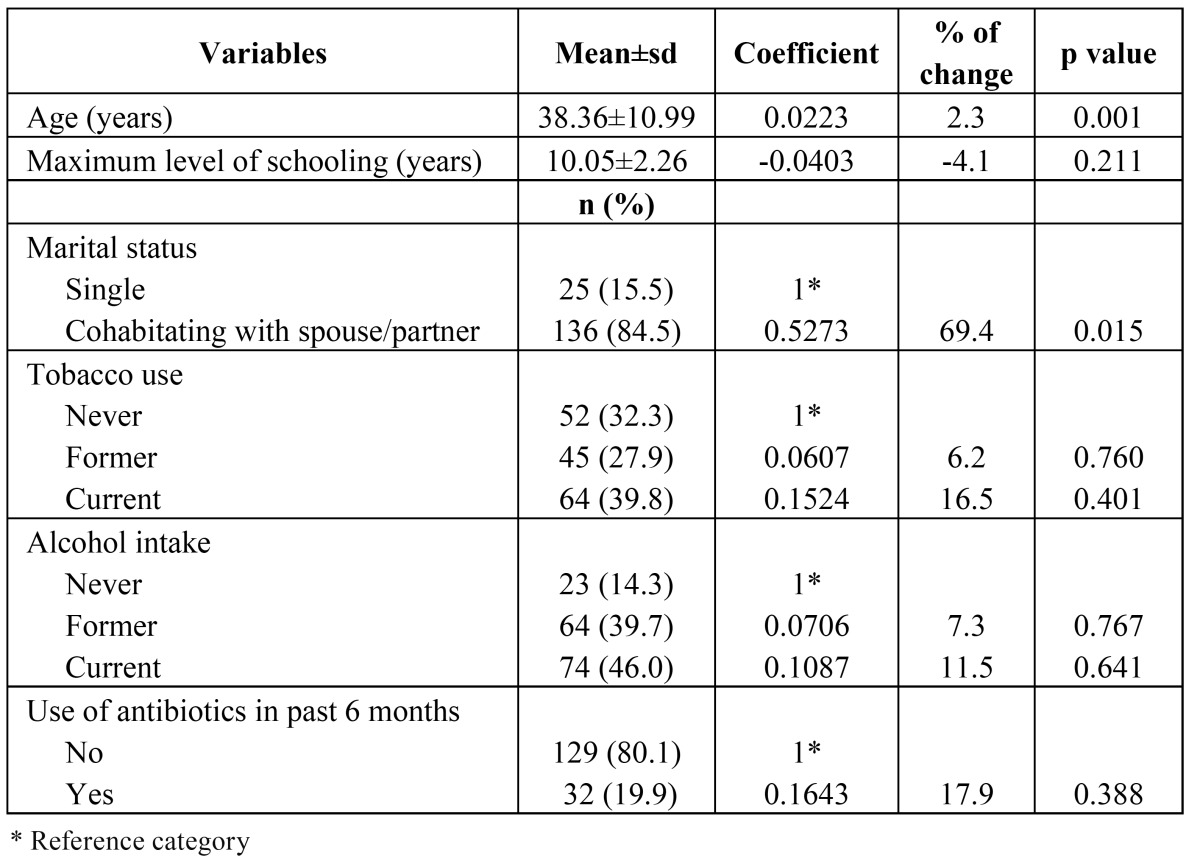
Descriptive analyses of socioeconomic and sociodemographic variables and others risk factors and bivariate analyses of negative binomial regression for gingival recession

**Table 2:**
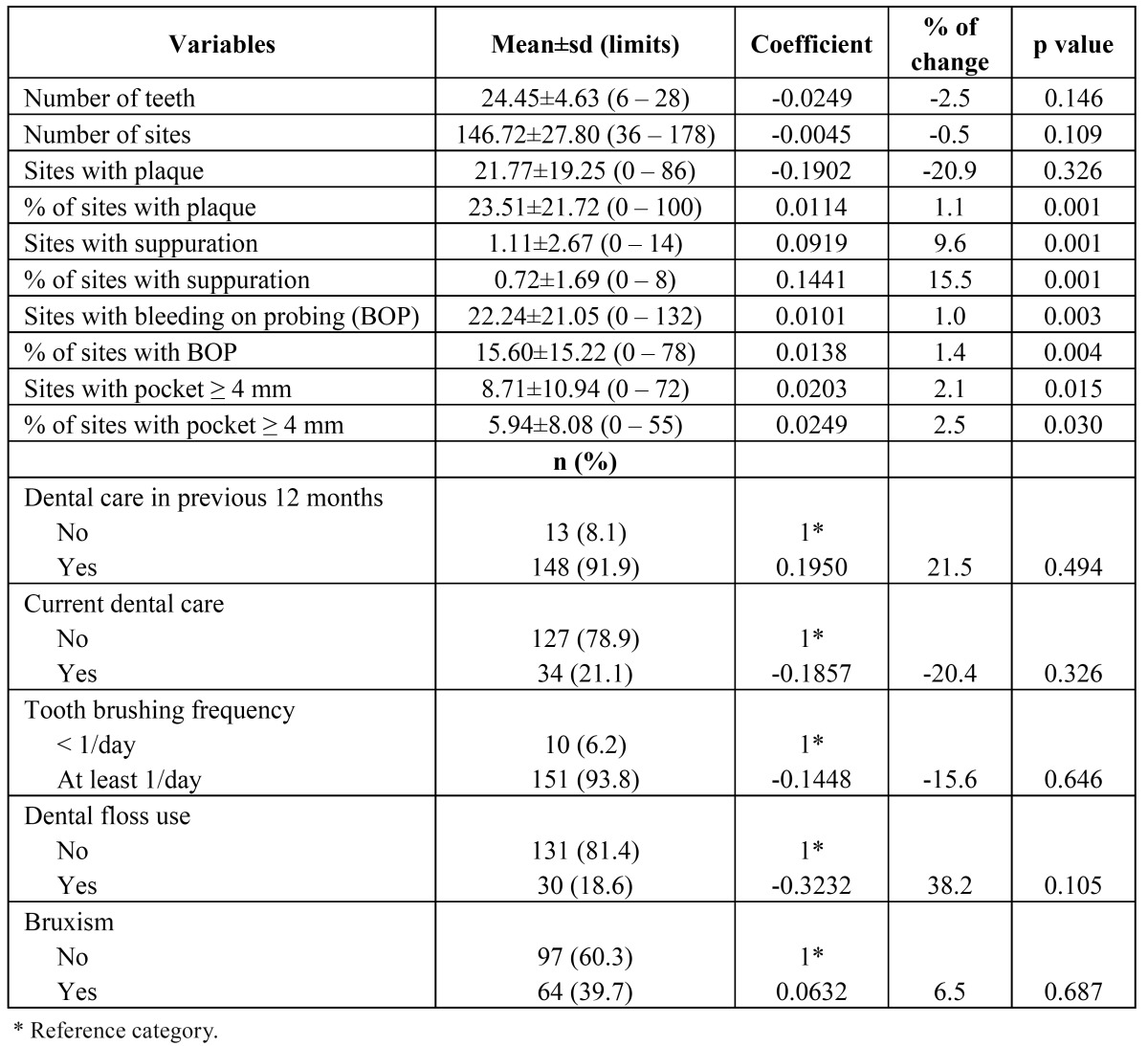
Descriptive analyses of dental variables and bivariate analyses of negative binomial regression for gingival recession.

Bivariate and multivariate results

Mean sites with periodontal pockets ≥ 4 mm without gingival recession was 2.25±4.10; for those sites associated with 1 to 5 mm, 6 to 10, 11 to 15, and 16 mm or larger, mean number of sites with pockets ≥ 4 mm were 9.26±13.39, 7.17±6.07, 11.19±9.48 and 14.79±12.97, respectively (Kruskall-Wallis p<0.001). We also ascertained that an increase in the number of sites with gingival recession was associated with an increase in the number of sites with pockets (non-parametric test for trend p<0.001).

Socio-demographic and socioeconomic characteristics and others risk factors were compared with the number of sites with gingival recession using the negative binomial regression model in a bivariate form. Only age and marital status had significant differences ( Table 1). ( Table 2) shows the results incorporating clinical variables and behavioral variables. The percentage of sites with plaque; sites and percentage of sites with suppuration; sites and percentage of sites with BOP; sites and percentage of sites with pockets ≥ 4 mm; were all associated with the number of sites with gingival recession. No behavioral variable was significantly related to the outcome variable.

To gauge the association between independent variables and gingival recession we used the negative regression model ( Table 3). In the adjusted negative binomial regression model we observed that for each year of age, the expected mean number of sites with recession increased 2.9%. This feature was dwarfed by the finding that among those study participants who lived with a partner in a marital relationship, the expected mean number of sites with recession increased 75.9%. Finally, the expected mean number of sites with recession increased 1.0% and 13.0% for each percentage point increase in sites affected by dental plaque, and sites with suppuration, respectively.

**Table 3:**
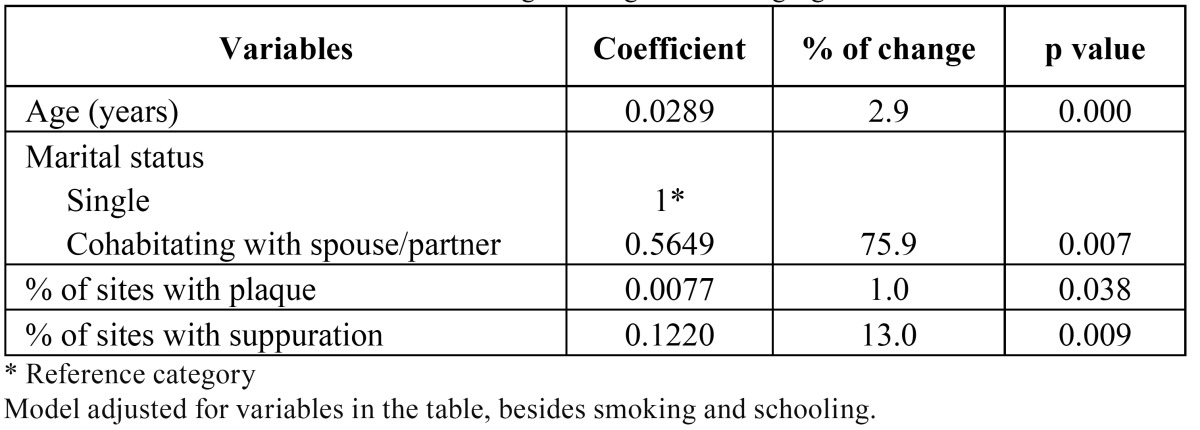
Multivariate model of binomial negative regression for gingival recession.

## Discussion

This study aimed to evaluate clinically gingival recession and to identify associated variables in a population of adult men. We found a prevalence of gingival recession of almost 90%, with an average of nearly 7 sites affected in each subject. Comparisons with other studies may be problematic mainly because of the varying definition of “case”: some authors consider gingival recession only if the exposed root is equal to or greater than 1 mm (20,21), between 1 and 2 mm (22), or 3+ mm (20). Furthermore, in the present study we measured six sites per tooth, while other researchers reported findings at the tooth level rather than by site: for example, Toker & Ozdemir (21) and Sarfati et al. (23) measured the gingival recession on the buccal site of each tooth. Similarly, differences in prevalence may be ascribable to certain characteristics of the study samples; e.g., age, sex, etc. In this specific regard we found a 87.6% prevalence of subjects with at least one site with gingival recession. Sarfati et al. (23) found similar prevalence (84.6%) in France among subjects 35-65 of age. Albandar & Kingman (20) used a sample of 9,689 Americans (30 to 90 years of age) and projected that 23.8 million people have 1+ tooth surfaces with gingival recession 3+ mm. They also found that the prevalence of 1+ mm recession was 58%. Mumghamba et al. (21) reported 33.6% gingival recession (1+ mm) prevalence in Tanzanian women. Murtomaa et al. (6) distinguished gingival recession prevalence (1+ mm) between women (69%) and men (49%) in Finland (25-26 years of age) .

Gingival recession can be localized or generalized, and be associated with one or more surfaces. Typically, gingival recessions are asymptomatic and develop slowly. Age is a variable that several authors have found associated with gingival recession. Like us, other authors (20,21,24) observed that with increasing age, the number of sites (or the risk to develop sites) with gingival recession also increased. Similar trends have often been found in other indicators connoting periodontal conditions. At the end of the day, however, our initial findings suggesting a correlation between the number of sites with gingival recession and with periodontal pockets were found to be unsubstantiated: the final multivariate model showed that such association was non-significant.In terms of marital status, a body of literature that establishes a link between this variable and gingival recession is largely absent. To complicate further such scenario, we found that those study participants with a couple had the largest number of sites with gingival recession. One might well postulate that the inverse relation would be observed as we know that married people or people with a steady partner have better oral health than people without a partner, or who are divorced or widowed (25). Interestingly, other studies have found that marital/partner status is associated with other periodontal health indicators: e.g., Chiou et al. (26) noted that according to a community periodontal index, married and divorced/widowed nonsmokers tended to have poorer periodontal health than single subjects. This relationship disappeared in smokers. Similar results were reported by Coelho et al. (27) in that married subjects had the worst periodontal condition; our observations seem to agree with other dental outcomes (28,29).

Traditional thinking postulates that risk indicator variables for periodontal disease are closely related to gingival recession; however our results only supported a link between gingival recession, and extent of plaque and extent of suppuration. Authors as Mumghamba et al. (21), Toker & Ozdemir (22), and Sarfati et al. (23) have found that biofilm, gingival bleeding, and presence of calculus, were associated with gingival recession on buccal sites – suggesting that some risk factors for gingival recession are similar to traditional periodontitis risk factors. None of these studies showed an association between gingival recession and suppuration.

A number of studies have confirmed a relationship between smoking and gingival recession (5); our data analyses did not, and while this is counterintuitive at first impression, we should keep in mind that the overall prevalence of smoking and former smoking were considerable. This is not altogether unexpected since this study group is a (generally) low SES population and also the tobacco use patterns in Mexico have not evolved in the same way that some developed countries populations have. We also failed to identify the expected relationships with alcohol use or bruxism. We found, however, the expected increase in former tobacco and alcohol users (6% and 7%, respectively) compared with nonusers, and with current users (tobacco and alcohol 16.5% and 11.5, respectively). Lack of associations between these factors and the gingival recession outcome might have resulted from possible low power to detect differences; at a minimum, the present scenario ought to be used in calculating from a better informed perspective the simple sizes in future studies in Mexico, given the current levels of alcohol and tobacco use, and the experience of periodontal conditions.

The present study has certain limitations that should be taken into account the place the findings in their appropriate context. Its design (cross-sectional study) makes it difficult to establish a cause-effect relationship because of the uncertainty from temporal ambiguity. The study population was made up exclusively of male participants: to our knowledge, the study is the first report on periodontal health aspects of Mexican policeman (an almost exclusively male occupation in the country) and specifically on gingival recession in Mexico. The study was carried out in an under served population from a dental services perspective who is at high risk of developing oral and other chronic diseases; supporting the strengths of the study design is the fact that the study subjects are a fairly homogeneous population group whose gingival changes are unlikely to have been modified by much periodontal (or, for that matter, dental) care. In conclusion, despite population idiosyncrasies, our solid methodology and analyses offered simple trends in terms of the association between gingival recession and sociodemographic and clinical parameters, in a geographic location about which little published information on gingival recession has hitherto been available.
